# No personality without experience? A test on *Rana dalmatina* tadpoles

**DOI:** 10.1002/ece3.1804

**Published:** 2015-11-28

**Authors:** Tamás J. Urszán, László Z. Garamszegi, Gergely Nagy, Attila Hettyey, János Török, Gábor Herczeg

**Affiliations:** ^1^ Behavioural Ecology GroupDepartment of Systematic Zoology and EcologyEötvös Loránd UniversityPázmány Péter sétány, 1/cBudapestH‐1117Hungary; ^2^ Department of Evolutionary EcologyEstacion Biologica de Donana – CSICc/Americo Vespucio, s/nSeville41092Spain; ^3^ Lendület Evolutionary Ecology Research GroupMTA ATK NÖVIHerman Ottó út 15Budapest1022Hungary

**Keywords:** Animal personality, behavioral syndrome, environment, evolution, plasticity

## Abstract

While the number of studies reporting the presence of individual behavioral consistency (animal personality, behavioral syndrome) has boomed in the recent years, there is still much controversy about the proximate and ultimate mechanisms resulting in the phenomenon. For instance, direct environmental effects during ontogeny (phenotypic plasticity) as the proximate mechanism behind the emergence of consistent individual differences in behavior are usually overlooked compared to environmental effects operating across generations (genetic adaptation). Here, we tested the effects of sociality and perceived predation risk during ontogeny on the strength of behavioral consistency in agile frog (*Rana dalmatina*) tadpoles in a factorial common garden experiment. Tadpoles reared alone and without predatory cues showed zero repeatability within (i.e., lack of personality) and zero correlation between (i.e., lack of syndrome) activity and risk‐taking. On the other hand, cues from predators alone induced both activity and risk‐taking personalities, while cues from predators and conspecifics together resulted in an activity – risk‐taking behavioral syndrome. Our results show that individual experience has an unequivocal role in the emergence of behavioral consistency. In this particular case, the development of behavioral consistency was most likely the result of genotype × environment interactions, or with other words, individual variation in behavioral plasticity.

## Introduction

Studying behavioral consistency has recently become a central topic of evolutionary behavioral ecology, aiming to understand the evolutionary and developmental mechanisms behind consistent individual differences in behavior. Behavioral consistency can be approached and quantified at two levels (Garamszegi and Herczeg 2012; Jandt et al. 2014; Urszán et al. 2015): First, individuals can consistently differ in certain behaviors (in aggression, for instance), and second, individuals can consistently differ across two or more functionally different behaviors (across aggression, exploration, and risk‐taking, for instance). The first form of behavioral consistency is statistically approached *via* repeatability, and called animal personality, while the second is approached *via* correlation, and called behavioral syndrome (Garamszegi and Herczeg 2012; Jandt et al. 2014; Urszán et al. 2015). It is important to note that pure phenotypic correlations between behavioral traits do not automatically prove the presence of behavioral syndromes, because syndromes are formed by between‐individual correlations and not within‐individual correlations (Dingemanse and Dochterman 2013; Dingemanse and Réale 2013). This implies that behavioral syndromes only form between behaviors that represent animal personalities.

Animal personalities and behavioral syndromes have been observed in the wild in a wide range of taxa (Smith and Blumstein 2008; Garamszegi et al. 2012, 2013). The evolutionary and ecological implications of these phenomena were repeatedly addressed (Sih et al. 2004a,b; 2012; Bell 2007; Biro and Stamps 2008; Kortet et al. 2010; Wolf and Weissing 2012; Dochtermann and Dingemanse 2013), leading to numerous hypotheses aiming to explain the emergence of behavioral consistency (Stamps 2007; Wolf et al. 2007; Dingemanse and Wolf 2013; Kight et al. 2013; Sih et al. 2015). Behavioral consistency might seem maladaptive at first glance. Behavior is often considered as the most plastic phenotypic trait (e.g., West‐Eberhard 2003), potentially allowing for permanent optimization following the temporal and spatial environmental variation. However, animal personality and behavioral syndromes can severely limit individual behavioral plasticity, either by decreasing individual behavioral repertoire (animal personality) or by linking functionally different behaviors (behavioral syndromes). Further, behavioral syndromes might not only limit behavioral flexibility by decreasing plasticity, but they have the potential to limit also the independent evolution of functionally different behaviors (Dochtermann and Dingemanse 2013; ).

There are two general hypotheses proposed to explain behavioral consistency (Bell 2005). According to the “constraint” hypothesis, behavioral consistency arises from underlying proximate mechanisms that are difficult to decouple through evolutionary time. Such mechanisms include a single hormone affecting multiple behaviors (Ketterson and Nolan 1999; Bell 2005), genetic linkage and pleiotropy (Dingemanse et al. 2007; Dochtermann and Dingemanse 2013), and physiological effects (Gosling 2001; Garamszegi et al. 2013). Quantitative genetic studies on behavioral traits imply the presence of genetic background of behavioral consistency and support the “constraint” view (Sih et al. 2004b; van Oers et al. 2005; van Oers and Mueller 2010; Dochtermann and Dingemanse 2013). On the other hand, the “adaptive” hypothesis states that behavioral consistency is a result of adaptation to the prevailing environment (Sih et al. 2004a; Bell 2005). However, because behavioral consistency is a group‐level phenomenon (repeatability in animal personality, correlation in behavioral syndrome), tests for local adaptation usually rely on population comparisons. Such studies have found that populations of the same species show the presence or absence patterns of behavioral syndromes congruent with the strength of predation risk, supporting the adaptive hypothesis (Bell 2005; Dingemanse et al. 2007).

Adaptive behavioral variation might emerge not only *via* genetic adaptations, but also *via* long‐lasting direct environmental induction, that is, phenotypic plasticity (the ability of a single genotype to produce different phenotypes induced by environmental variation, West‐Eberhard 2003). There is evidence that (1) behavioral syndromes can be induced in predator‐naive individuals originally lacking the syndrome by exposing them to predation risk (Bell and Sih 2007), (2) environmental complexity in general is a key factor in the formation of behavioral consistency (Sweeney et al. 2013; Bengston et al. 2014; Härkönen et al. 2014), and (3) small perturbations during ontogeny might affect behavioral consistency at later stages (Urszán et al. 2015). However, studies investigating the role of the environment in the development of behavioral consistency within and across behaviors (animal personality and behavioral syndrome, respectively) in manipulative experiments are scarce at best.

**Figure 1:**
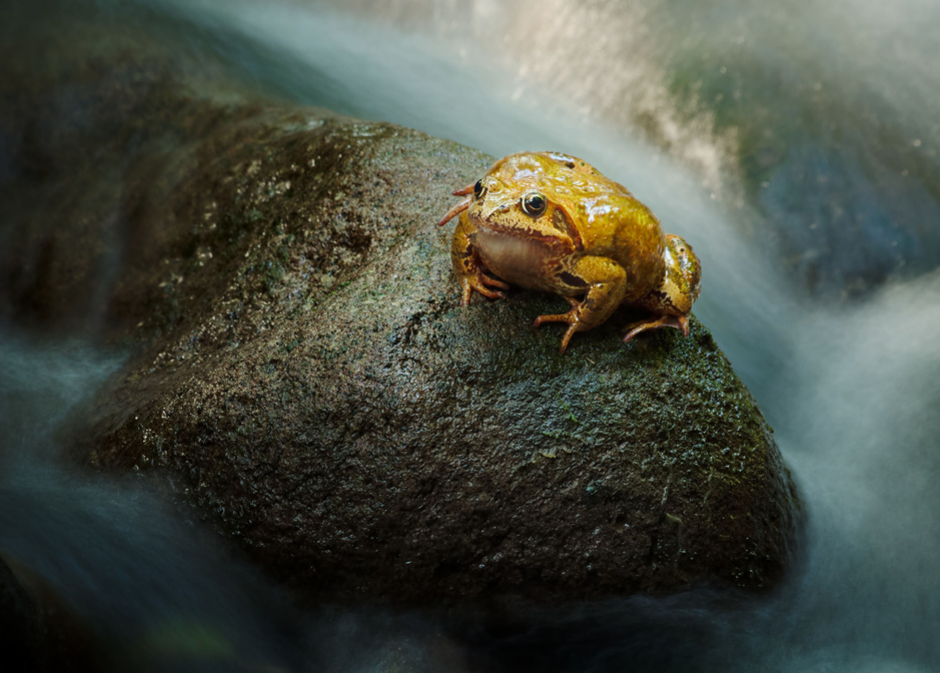
Adult agile frog (*Rana dalmatina*). Photograph credit goes to Miklós Laczi.

Here, we aimed to investigate the role of ecologically relevant environmental stimuli in the emergence of behavioral consistency. We hypothesized that a group‐level phenomenon like behavioral consistency, manifesting in the form of animal personality or behavioral syndrome, is not purely genetically determined, but needs ecologically relevant environmental stimuli to emerge. If our hypothesis was true, animal personality and behavioral syndromes should not emerge across individuals that were reared in isolation from conspecifics, predators, or parasites, while receiving food and water ad libitum. As previous studies showed that amphibians provide good models for behavioral consistency research (Sih et al. 2003; Wilson and Krause 2012; Urszán et al. 2015), we studied agile frog (*Rana dalmatina* Fitzinger in Bonaparte, 1839; Fig 1) tadpoles. We tested our hypothesis by assessing activity and risk‐taking three times in tadpoles reared under different treatments. Tadpoles were reared from hatching in a full‐factorial common garden experiment in laboratory, with two levels of predation (predatory cues present/absent) and group (conspecifics present/absent) treatments. Besides testing for the presence of behavioral consistency, we also assessed how our treatments affected mean behavior to see whether it had the expected effects on behavior in general. This could have been particularly important for interpreting negative results (i.e., lack of treatment effects on behavioral consistency). We predicted that the presence of predatory cues will decrease activity and risk‐taking, while the presence of conspecifics will weaken the predatory effects by diluting the perceived *per capita* predation risk.

## Materials and Methods

### Field sampling and rearing

*Rana dalmatina* eggs were collected from a pond on the Island of Szentendre in the vicinity of Szigetmonostor (47°40′40.77″ N, 19°5′31.47″ E). Located on the floodplain of the Danube, this pond exposes tadpoles to numerous invertebrate and vertebrate predators. Forty clutches were sampled between 21 March and 8 April in 2013. We randomly selected 120 eggs from each clutch and divided them into four groups of 30 eggs kept in 1.5‐L plastic containers (20.6 × 14.6 × 7.5 cm, length, width, height, respectively) filled with 0.8 L of reconstituted soft water (RSW, APHA 1985). Temperature was set to 19°C, and a 12/12‐light–dark photoperiod (light period lasted from 0800 to 2000 h) was provided during the whole experiment. As predators, we have used late instars dragonfly (*Anax imperator* Leach, 1815) larvae collected from the sampled breeding pond of *R. dalmatina* and a juvenile pike (circa 8 cm long; *Esox lucius* Linnaeus, 1758) acquired from a fishery. Note that pike was also observed in the study pond. The dragonfly larvae were kept in plastic cups filled with 0.5 L of RSW, while the pike was kept in a plastic container with 8 L of RSW. During the course of the experiment, the dragonfly larvae were fed with tadpoles. We fed the dragonfly larvae every third day in a shifted way so we could sample water from eating, satiated, and hungry larvae each day. The juvenile pike was fed multiple tadpoles daily.

Treatments started when the tadpoles hatched. Note that we had four replicates with 30 tadpoles in each, from every clutch. These within‐clutch replicates were assigned to the four factorial treatments (see below) randomly, resulting in 40 replicates (one per clutch) for each treatment.

- In the “naïve” treatment (no predator, no conspecifics), we randomly selected one healthy tadpole from every clutch and reared it alone by removing the rest of the tadpoles from the container. We administered 40 mL of control water twice a day, consisting of only clear RSW. For water administration, we used a 60‐mL syringe here, and in the other treatments as well.
- In the “predation” treatment (predator, no conspecifics), we randomly selected one healthy tadpole from every clutch and reared it alone by removing the rest of the tadpoles from the container. We administered 40 mL of stimulus water twice a day, consisting of 20 mL RSW containing olfactory cues from the predators (taken in a 1:1 ratio from dragonfly larvae and the pike) and 20 mL clear RSW.
- In the “conspecifics” treatment (no predator, conspecifics), we randomly selected five healthy tadpoles from every clutch and reared them in group by removing the rest of the tadpoles from the container. We administered 40 mL of stimulus water twice a day, consisting of 20 mL RSW with conspecific odor (see below) and 20 ml clear RSW.
- In the “predation and conspecifics” treatment (predator, conspecifics), we randomly selected five healthy tadpoles from every clutch and reared them in group by removing the rest of the tadpoles from the container. We administered 40 mL of stimulus water twice a day, 20 mL RSW containing olfactory cues from the predators (taken in a 1:1 ratio from dragonfly larvae and the pike) and 20 mL RSW with conspecific odor (see below).

Altogether, 40 replicates per treatment, that is, 40–40 individuals for the “control” and “predation” groups and 200‐200 individuals for the “conspecifics” and “predation and conspecifics” groups were included in the experiment, using the forty clutches to maximize genetic diversity within treatment. We note that we did not (and since only one family member was present in any given treatment, could not) aim to test for family effects. As the experimental animals were planned to be tested in several ways, we utilized only half of them (ideally *N* = 20 in each treatment group) for the experiment reported here. Note that individuals were chosen randomly, hence, not the same 40 clutches were represented in the treatments. Mortality in the early developmental stage, clear deformities in some individuals, and some recording errors all contributed to the loss of a few specimens. At the end, we could use 18 individuals in the control treatment; 20 individuals in the predation only treatment; 18 in the conspecifics only treatment; and 17 in the predation and conspecifics treatment. Tadpoles not used in the experiment were used as a source of conspecific odor and as food for predators and were kept in large containers filled with RSW. All tadpoles were fed with minced and boiled spinach ad libitum, food being administered 3 h before the end of the daily light period. Water was changed every four days.

### Behavioral assays

Individual development was followed on a daily basis. When individuals chosen for the experiment have reached stage 32–36 (Gosner 1960; early stages of toe development), we performed behavioral assays. In group‐reared tadpoles, a single individual was selected randomly and all other tadpoles were removed from the rearing container. For all individuals entering behavioral assays, water was changed and treatment water was administered as usual. The following day we began trials, which lasted three days. We assessed two different personality traits (Réale et al. 2007; Garamszegi et al. 2013): activity and risk‐taking.

The behavior of the tadpoles was recorded with webcams using the open source Dorgem software (Fesevur, http://dorgem.sourceforge.net/). Before each behavioral test, we administered stimulus water. Activity was assessed between 1000 and 1030 h and then risk‐taking between 1230 and 1305 h. We measured activity first, because it is a noninvasive measurement that did not affect the experimental individuals. Assessment of risk‐taking, on the other hand, included a potentially stressful stimulus (see below). Activity was estimated by measuring the distance moved in the familiar environment during 30‐min observational period with MATLAB (Hedrick 2008). Risk‐taking was estimated by latency to restart activity (time spent immobile) following a simulated attack. We used a fine paint brush (#00) to poke the tadpoles at the base of their tails to mimic a predator attack. Tadpoles responded to the stimulus with rapid escape behavior and subsequent immobility. Individuals that remained immobile for the entire 35 minutes of the observational period were assigned the maximum score of 2100 sec.

### Statistical analysis

Both behavioral variables were log_10_‐transformed to achieve better distribution. To test whether the treatment affected the mean behavior expressed in the different groups, we ran two general linear mixed models (GLMMs) on the different behaviors with the treatments and their interaction as fixed effects and individual as a random effect using restricted maximum likelihood estimation available in the lme4 package (Bates et al. 2011) in the R statistical environment (R Developmental Team 2014). To obtain group‐specific repeatability estimates for the two traits, we fitted GLMMs separately on each subset of the data that corresponded to different treatments by including only intercept in the fixed part of the models. We extracted variance components from these models and calculated the proportion of the between‐individual variance relative to the total variance (Nakagawa and Schielzeth 2010).

Because phenotypic behavioral correlations are not necessary reflecting between‐individual differences, one needs to statistically decompose between‐ and within‐individual correlations to be able to correctly judge behavioral syndromes (Dingemanse et al. 2012; Dingemanse and Dochterman 2013). Therefore, to compare the presence/absence/strength of behavioral syndromes across the experimental groups, we performed bivariate mixed models using activity and risk‐taking jointly as response variables and assuming that these two traits were assayed at the same time (i.e., right after each other). The model included only random intercepts for the factor “Individual” and was run separately for each treatment group. From these models, we calculated the between‐individual correlations of traits based on the estimated variance and covariance components as suggested by Dingemanse and Dochterman (2013). These models were fitted by the MCMCglmm package (Hadfield 2010). We used uninformative, inverse gamma priors and relied on long (1 300 000 with 300 000 burnin) iterations. Each model was run at least four times to verify the stability of results. From these Markov chains, we took 1000 samples, over which we calculated the posterior mode (using kernel density estimation) to obtain the parameter estimates of interest, and the highest posterior density intervals to obtain the 95% credibility intervals around them. To assess the importance of the effect of within‐individual correlations, as a contrast analysis, we calculated the phenotypic correlations between traits using classical approaches, in which the correlation between traits was estimated based on the individual‐specific means. However, we provide these results merely for illustration, as for interpretations about between‐individual correlation (behavioral syndromes), we rely on the outputs of the bivariate mixed models.

## Results

### Behavioral types

**Figure 2:**
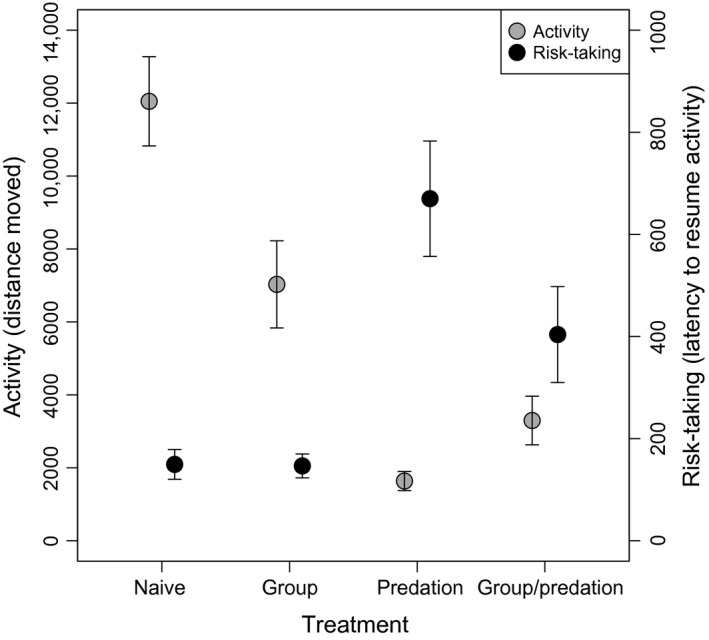
Behavioral differences induced by the group and predation treatments in agile frog (*Rana dalmatina*) tadpoles. Activity is represented by distance moved (mm) during the observation period. Risk‐taking is estimated by the latency (sec) to restart activity after a simulated attack. Means + standard errors are shown.

The GLMM revealed that predation treatment had an effect on activity (predation: *F*_1,70_ = 27.33, *P *<* *0.001; group: *F*_1,70_ = 0.16, *P *=* *0.69; predation × group: *F*_1,69_ = 3.75, *P *=* *0.057): It decreased in tadpoles developing in the presence of olfactory cues from predators (Fig. 2). The marginally significant interaction showed a trend where tadpoles under perceived predation risk showed higher activity in groups than alone. The GLMM on risk‐taking revealed a similar pattern (predation: *F*_1,70_ = 35.27, *P *<* *0.001; group: *F*_1,70_ = 2.71, *P *=* *0.10; predation × group: *F*_1,69_ = 2.66, *P *=* *0.11): Tadpoles developing in the presence of olfactory cues from predators decreased their risk‐taking (Fig. 2).

### Personality and behavioral syndromes

**Figure 3:**
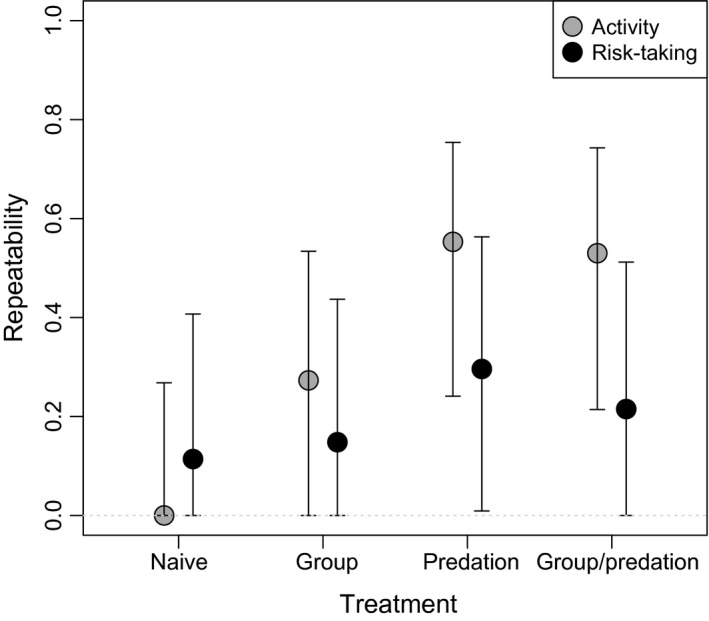
Differences in single behavior consistency, that is, animal personality, induced by the group and predation treatments in agile frog (*Rana dalmatina*) tadpoles. Repeatabilities + 95% confidence intervals are shown.

Repeatability estimates as obtained separately for each treatment group revealed that activity had a modest (<0.3) repeatability that was associated with a 95% confidence interval that included zero in the “naïve” and “conspecifics” treatments, but when the “predation” treatment was applied (alone or in combination with conspecifics), considerably higher (>0.5) repeatabilities emerged with 95% confidence intervals that were far away from zero (Fig. 3). Repeatability estimates for risk‐taking covered smaller ranges that were systematically below 0.5 and spanned down to zero. The only exception was the “predation” treatment group, which showed marginally significant nonzero repeatability for the trait from the given data (Fig. 3).

**Figure 4:**
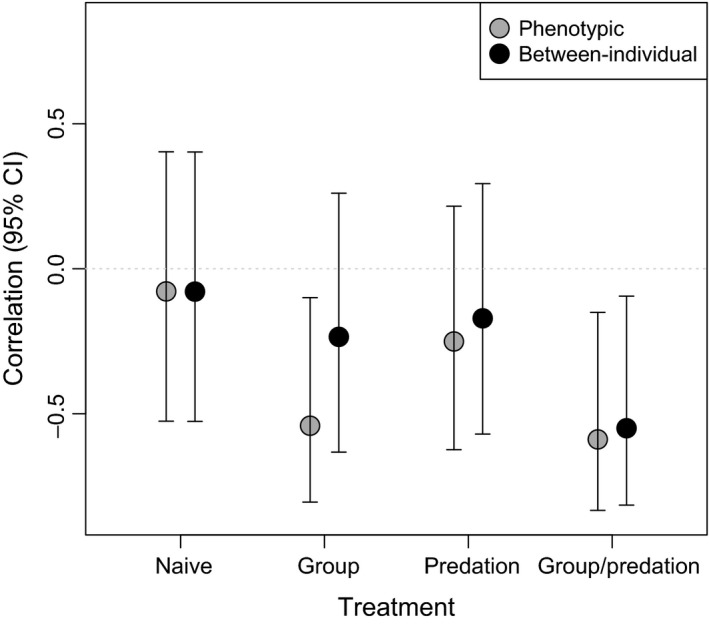
Differences in across behavior consistency, that is, behavioral syndrome, induced by the group and predation treatments in agile frog (*Rana dalmatina*) tadpoles. Note that between‐individual correlations are indicative of behavioral syndromes, and phenotypic correlations are shown only for illustrative purposes. Correlation coefficients + 95% credibility intervals are shown.

When taking into account within‐individual variance and covariance of traits using bivariate mixed models, we detected nonzero between‐individual correlation in the “predation and conspecifics” treatment group only (Fig. 4). This pattern indicated a strong negative relationship (<−0.5) between our variables, which suggests a positive relationship between activity and risk‐taking: Those individuals that moved longer distances undisturbed in a familiar environment had shorter latencies to restart their activity after a simulated attack. Note that these tendencies could also be detected in the other treatment groups (in fact, phenotypic correlation would show a significant effect for the “conspecifics” treatment group too), but with considerably smaller magnitudes (Fig. 4).

## Discussion

Predation and intraspecific competition are major ecological factors that often induce phenotypic plasticity in numerous traits (e.g., Miner et al. 2005; Callahan et al. 2008). Predation is a key factor affecting fitness (e.g., Roff 1992; Tollrian and Harwell 1999), while being in a group has both costs and benefits. For instance, resource limitation and stress arising from agonistic encounters can incur costs, while decreased *per capita* predation risk can be a benefit of grouping (Pitcher and Parrish 1993; Krause and Ruxton 2002). In the present paper, we found that both predation and group living had considerable effects on *R. dalmatina* tadpoles' behavior. First, as expected (e.g., Tollrian and Harwell 1999), perceived predation risk decreased activity and risk‐taking, and there was a trend of weakening the effect of perceived predator risk by group living. Second, and most importantly, we were unable to find significant evidence for behavioral consistency both within and across the studied behaviors in predation‐ and conspecifics‐naïve tadpoles. Meanwhile, we could detect repeatabilities and a between‐individual correlation that were statistically differentiable from zero in tadpoles that had been reared under predation pressure and/or with a group of conspecifics. In other words, only tadpoles exposed to ecologically relevant environmental stimuli developed animal personality or behavioral syndrome.

Regarding activity, perceived predation risk (irrespective of the group treatment) induced significant behavioral consistency. Repeatability estimates in these groups fall within the range between 0.21 and 0.74, which can be seen as similar or even higher estimates than that of the typical behavioral traits with the mean value of 0.37, as reported in a meta‐analysis (Bell et al. 2009). Risk‐taking was significantly repeatable (i.e., statistically distinguishable from zero with the available data) in the predation treatment (*R* = 0.01–0.56). In the predation and conspecifics treatments, we detected no significant repeatability, but there was a trend of emerging repeatability among some individuals (*R* = 0.0–0.51). These values can be interpreted as representing moderately to weakly consistent personalities (Bell et al. 2009). For both activity and risk‐taking, predation seems to be the key stimulus to induce significant individual variation in behavior. Between‐individual correlation between activity and risk‐taking was only present in the predation and conspecifics treatment, with an effect size above 0.5, which translates to a strong behavioral syndrome considering the mean value of 0.2 reported in a meta‐analysis of phenotypic behavioral correlations (Garamszegi et al. 2012). There was a trend for phenotypic correlation (including also the within‐individual component) with an effect size above 0.5 in the conspecifics treatment, but this cannot be seen as an indication of behavioral syndrome (Dingemanse et al. 2012; Dingemanse and Dochterman 2013).

In our view, the main question in association with behavioral consistency is why it exists in the first place. Intuitively, it is a phenomenon that limits both the plasticity (*via* animal personalities and behavioral syndromes) and the evolution (*via* behavioral syndromes) of behavior. The two main hypotheses invoked to explain behavioral consistency are the constraint hypothesis, assuming a limiting proximate mechanism, and the adaptive hypothesis, assuming a selective ultimate mechanism (Bell 2005; Dingemanse et al. 2007; Han and Brooks 2013). While these hypotheses typically focus on behavioral syndromes, they are equally relevant for animal personalities. Several studies reporting significant heritabilities of behavior or genetic correlations between behaviors (e.g., van Oers et al. 2005; Dochtermann and Dingemanse 2013) support the constraint view. The adaptive hypothesis has also been worked out in detail from different aspects (e.g., Stamps 2007; Wolf et al. 2007; Dingemanse and Wolf 2010). However, testing these hypotheses is not straightforward. As behavioral consistency *per se* cannot be studied at the individual level, which is a prerequisite of standard evolutionary studies, one has to rely on comparing populations from different environments. Such population comparisons yielded mixed results. Some rejected the constraint hypothesis (Bell 2005), some supported the adaptive hypothesis (Dingemanse et al. 2007), some found patterns congruent with the constraint hypothesis (Pruitt et al. 2010), and some found mixed results (Brydges et al. 2008). Further, even if some environments generate behavioral syndromes, their correlation structure can be different (Royauté et al. 2014).

The role of ontogenetic experience in shaping behavior has been emphasized and demonstrated lately (Dingemanse et al. 2009; Stamps and Groothuis 2010a,b; Rodel and Monclus 2011; Butler et al. 2012; Carere and Maestripieri 2013). Patterns can even emerge in the form of unwanted side‐effects of laboratory manipulations (Ruiz‐Gomez et al. 2008; Urszán et al. 2015). Here, we experimentally demonstrated that ontogenetic experience with ecologically relevant environmental stimuli is necessary for the development of behavioral consistency in our species. This result is hard to interpret within the constraint *vs*. adaptive framework because we studied only one population, but we can reject the existence of a proximate constraint that will result in behavioral consistency under any circumstances. The importance of direct environmental effects for the emergence of behavioral syndromes has been suggested also in fish (Bell and Sih 2007) and in spiders (Sweeney et al. 2013). In the latter study, only spiders reared in the natural environment developed a behavioral syndrome. Because the natural environment did not induce significant mortality and young field‐reared spiders lacked the syndrome, the authors concluded that phenotypic plasticity alone might be enough to create behavioral consistency.

Considering that we were not able to find statistical evidence for animal personality or behavioral syndrome in predator‐ and conspecifics‐naïve tadpoles, we suggest that between‐individual differences in behavior are unlikely to emerge as a pure consequence of the underlying genetic variation and that there is a fundamental role for environmental effects in their emergence. In our study, perceived predation risk seemed to be the key factor inducing animal personality. The predation treatment was planned to be uniform for each individual, and hence, predation‐induced personality is most likely a result of an interaction between the individual genotypes and the environment. In other words, the detection of individually varying behavioral types as answers to a standardized stimulus are likely to stem from individual variation in behavioral plasticity (Dingemanse and Wolf 2013). Group living together with perceiving predatory threat somewhat weakened the between‐individual differences in the single behaviors, perhaps by diluting the perceived *per capita* risk. However, the picture was completely different for the between‐individual correlation indicating a behavioral syndrome (Dingemanse et al. 2012; Dingemanse and Dochterman 2013): Only the treatment where both predatory cues and conspecifics were presented during development induced its emergence. As opposed to the uniform predation treatment, group treatment is expected to provide different stimuli for individuals having different ranks in the hierarchy, and thus it had the potential to induce individual variation without genetic variation. Properly explaining the mechanism induced by the two treatments' interaction that results in the strong link between functionally different behaviors would be overly speculative. However, it is clear that (1) animal personality and behavioral syndrome can have different developmental routes and thus (2) probably different functions and adaptive values too. This result reinforces the need to separate the two levels of behavioral consistency (Garamszegi and Herczeg 2012; Jandt et al. 2014).

Within‐individual behavioral correlations are not considered as behavioral syndromes (Dingemanse et al. 2012), but can result in phenotypic behavioral correlations alone and thus deserve full biological consideration. These correlations can be seen as results of correlational plasticity within individuals (Dingemanse et al. 2010; Dingemanse and Réale 2013). Such patterns have been reported for instance in birds under starvation (Lima and Dill 1990). In our group treatment without predatory cues, a strong phenotypic correlation emerged between activity and risk‐taking. As between‐individual correlation was absent, this must have been a result of the within‐individual correlations. Note that there were no animal personalities present in this treatment either. Phenotypic behavioral correlation between behaviors that did not represent personality traits (i.e., were not repeatable) was reported in fish earlier (Bell and Stamps 2004). This means that while individuals were inconsistent in their behavioral ranks within the studied group, their behaviors became linked and changed together as a result of our group treatment. One possible explanation for this is that the hierarchy within the groups was unstable or nonlinear, and thus, individual state varied between the days of the assays. The emergence of within‐individual correlations (note that it was absent in the naïve treatment) warrants future investigations to better understand the way it develops and its effect on fitness.

In a previous laboratory study on the same population of the same species, we found that “naïve” tadpoles had moderate but significant levels of consistency in activity and exploration (Urszán et al. 2015). However, that experiment was ran in another laboratory, individuals were housed in larger containers with a high contrast grid applied on their bottoms, compared to the smaller and plain containers of this study. In the previous study, we also found that even minor manipulations during standard behavioral assays in an early developmental stage can have profound effects on behavioral consistency seen in later ontogenetic stages. It seems that even overly simple environmental variation has the potential to induce at least some forms of behavioral consistency. These patterns point also to the difficulty of controlling environmental effects that can induce behavioral shifts and the emergence of behavioral consistency even in standardized laboratory experiments.

Taken together, our experiment showed that both animal personality and behavioral syndrome can be an induced response to ecologically relevant stimuli originating from conspecifics or predators in a *R. dalmatina* population experiencing high predation. These results lend support for the notion that ontogenetic experience may play a key role in the emergence of behavioral consistency. Hence, it is likely that even adaptive evolutionary patterns might only manifest in the form of gene – environment interactions. This means that the emphasis might be on individually variable behavioral plasticity instead of assuming rigid behavioral variation among individuals. Further studies are needed to separate the effects of genes, environment and their interaction on animal personalities and behavioral syndromes. Possible solutions include using laboratory animals with known genetic background and testing for environmental effects on presence/absence/strength of behavioral consistency at the group‐level (see Carere and Maestripieri 2013 for promising results obtained with laboratory rodents), or using individual‐level estimates of behavioral consistency (e.g., Herczeg and Garamszegi 2012; Stamps et al. 2012) and subject it to standard evolutionary testing (for recent examples, see Briffa 2013; Briffa et al. 2013; Westneat et al. 2014; Bridger et al. 2015).

## Conflict of Interest

None declared.
